# Empfehlung zu Vitamin D im Rahmen der COVID-19-Pandemie für Geriater*Innen und Hausärzt*Innen

**DOI:** 10.1007/s41975-021-00180-5

**Published:** 2021-02-18

**Authors:** Heike A. Bischoff-Ferrari, Reto W. Kressig, Christian Meier, Petra Stute

**Affiliations:** 1 Geriatrie, Universitätsspital Zürich, Zürich, Schweiz; 2 Universitäre Klinik für Akutgeriatrie, Stadtspital Waid & Triemli, Zürich, Schweiz; 3 Lehrstuhl Geriatrie und Altersforschung, Universität Zürich, Zürich, Schweiz; 4 Universitäre Altersmedizin FELIX PLATTER, Basel, Schweiz; 5 Klinische Professur für Geriatrie, Universität Basel, Basel, Schweiz; 6 Klinik für Endokrinologie, Diabetologie und Metabolismus, Universitätsspital Basel, Basel, Schweiz; 7 Endokrinologische Praxis, Osteologisches Universitätsforschungszentrum DVO, Basel, Schweiz; 8 Abteilung für Gynäkologische Endokrinologie und Reproduktionsmedizin, Frauenklinik, Inselspital, Friedbühlstrasse 19, 3010 Bern, Schweiz; 9 Schweizerische Gesellschaft für Gynäkologische Endokrinologie und Menopause, SGEM, Lausanne, Schweiz

## Ausgangslage

In der aktuellen Pandemiesituation sind volksgesundheitliche Massnahmen zur Risikoverminderung der COVID-19-Infektion dringend gesucht. Die Hinweise zu Vitamin D als mögliche präventive Massnahme häufen sich und obgleich die in einer idealen Welt geforderte Evidenz einer grossen Studie zu Vitamin D bei COVID-19-erkrankten Patienten aktuell aussteht, sind aus einer volksgesundheitlichen Sicht in der aktuellen Krisensituation umsichtige Regeln, gestützt auf eine umfassende Risiko-Benefit-Analyse, notwendig, um potenziell Menschenleben zu retten. Aus dieser Perspektive betrachtet sprechen aus volksgesundheitlicher Sicht mehr Argumente für eine präventive Gabe von 400 bis 1000 IE Vitamin D am Tag im Rahmen der COVID-19-Pandemie als dagegen.

Dieses Statement basierend auf neusten wissenschaftlichen Daten soll die aktuelle Kontroverse zu Vitamin D in der Laienpresse sachlich ergänzen und Geriater*Innen und Hausärzt*Innen unterstützen, die Aussagen in der Laienpresse einzuordnen.

## Was ist die wichtigste Evidenz für eine Empfehlung zur Vitamin-D-Supplementation in der aktuellen COVID-19-Pandemie-Situation?

Das wichtigste Argument für eine volksgesundheitliche Empfehlung zur präventiven Gabe von Vitamin D im Rahmen der aktuellen Pandemiesituation ist eine aktuelle Metaanalyse von 42 hochqualitativen klinischen Studien mit total 46.331 Studienteilnehmern und Studienteilnehmerinnen im Alter von 0 bis 95 Jahren. Ziel dieser Metaanalyse war herauszufinden, inwieweit eine Vitamin-D-Gabe das Risiko von akuten Atemwegsinfekten senken kann. Einschränkend bezogen auf die aktuelle Pandemiesituation ging es hier um akute Atemwegsinfekte jeglicher Art, also nicht speziell um COVID-19-verursachte akute Atemwegsinfekte. Das Resultat dieser Metaanalyse ist zudem noch nicht zur Publikation angenommen, aber bereits umfassend in einer Vorpublikation auf dem Internet einsehbar^1^.

Über alle 42 Interventionsstudien dieser Metaanalyse hinweg zeigt sich ein bescheidener Effekt mit einer signifikanten 9 %igen Verminderung der akuten Atemwegsinfekte unter Vitamin D (Odds Ratio [OR] = 0,91, 95 %-KI 0,84–0,99). Die Autoren weisen jedoch darauf hin, dass die Analyse aller Studien möglicherweise den Benefit von Vitamin D unterschätzt, weil eine grosse Heterogenität zwischen den Studien festgestellt wurde. Tatsächlich zeigte das Ergebnis der Studien, welche die heutige Empfehlung von täglich 400–1000 IE Vitamin D untersucht hatten, ein deutlicheres und homogeneres Ergebnis, mit einer Verminderung der akuten Atemwegsinfekte um 30 % (OR = 0,70, 95 %-KI 0,55–0,89). Und für 8 Studien in der täglichen Dosierung von 400 bis 1000 IE Vitamin D in einer Anwendung bis zu 12 Monaten war das Ergebnis am ausgeprägtesten und homogensten mit einer Verminderung der akuten Atemwegsinfekte um 42 % (OR = 0,58, 95 %-KI 0,45–0,75).

Die wöchentlichen oder monatlichen Bolusgaben von Vitamin D waren hingegen nicht wirksam in der Verminderung akuter Atemwegsinfekte, während die tägliche Gabe von Vitamin D das Risiko akuter Atemwegsinfekte unabhängig von der Dosierung um 25 % verminderte (OR = 0,75, 95 %-KI 0,61–0,93).

Aktuell findet eine Ergänzung dieser neusten Metaanalyse mit den Daten der DO-HEALTH-Studie statt [1]. DO-HEALTH untersuchte die zusätzliche Gabe von 2000 IE Vitamin D am Tag und zeigte eine signifikante 16 %ige Verminderung jeglicher Infekte, jedoch nur bei den jüngeren Teilnehmern und Teilnehmern im Alter von 70 bis 74 Jahren. In DO-HEALTH hatte der überwiegende Teil der Studienteilnehmer*Innen zu Beginn der Studie keinen Vitamin-D-Mangel und alle Studienteilnehmer*Innen durften die heutige Empfehlung von 800 IE Vitamin D am Tag zusätzlich zur Studienmedikation einnehmen.

Zusammenfassend bezüglich der Einordnung dieser neusten Metaanalyse ist deren Ergebnis aus volksgesundheitlicher Sicht in der aktuellen Pandemiesituation relevant, weil COVID-19 ein akuter viraler Atemwegsinfekt ist. Mit und im schlimmsten Fall ohne Ansprechen auf das COVID-19-Virus ist eine Reduktion jeglicher akuten Atemwegsinfekte um 30 % mit der Einnahme von 400 bis 1000 IE Vitamin D am Tag volksgesundheitlich hoch relevant. Im Rahmen einer Risiko-Benefit-Abwägung käme mit dieser Empfehlung zudem niemand zu Schaden, im Gegenteil, mit dieser Empfehlung könnten minimal andere virale und bakterielle akute Atemwegsinfekte gesenkt werden und vulnerable ältere Menschen mit Vitamin-D-Mangel hätten den belegten Benefit einer Risikoverminderung von Knochenbrüchen und Stürzen [2–6]. Bezüglich der Sicherheit ist Vitamin D in der täglichen Dosierung von 400 bis 1000 IE am Tag bereits in den präventiven Vitamin-D-Empfehlungen seitens des BAG (Federal Commission for Nutrition. Vitamin D deficiency: Evidence, safety, and recommendations for the Swiss Population. Expert report of the FCN. Zurich: Federal Office for Public Health, 2012) verankert, die zur Prävention ohne notwendige vorherige Testung des Blutspiegels zur Anwendung kommen, mit 97 % Sicherheit den Vitamin-D-Mangel korrigieren und Menschen ohne Vitamin-D-Mangel nicht gefährden [7].

## Welche Hinweise gibt es spezifisch zu Vitamin D und COVID-19?

Bezogen auf COVID-19 wurde in den vergangenen Monaten in der überwiegenden Anzahl publizierter *Kohortenstudien* beobachtet, dass Personen mit einem Vitamin-D-Mangel ein erhöhtes COVID-19-Risiko und eine erhöhte COVID-19-Mortalität aufweisen [8–12]. Kohortendaten belegen jedoch keine Kausalität und können wesentliche Schwächen aufweisen [13].

Dazu kommen *basiswissenschaftliche Studien*, die zeigen, dass Vitamin D den COVID-19-induzierten Zytokinsturm zu unterdrücken scheint [14, 15], als mögliche Erklärung, warum in verschiedenen Kohortenstudien ein höherer Vitamin-D-Blutspiegel mit einem geringeren Schweregrad der COVID-19-Erkrankung korreliert [8–12]. Bekannt ist, dass Vitamin D eine Rolle in der angeborenen und akuten Immunantwort spielt [16]. Entsprechend ist der Vitamin-D-Rezeptor (Andockstelle) auf vielen Zellen des Immunsystems verankert [16] und vermittelt zum Beispiel die Freisetzung von Entzündungsfaktoren (Zytokinen) durch sogenannte Fresszellen und trägt damit zu einer Hochregulation von antimikrobiellen Substanzen bei, die eine antivirale Wirkung haben [17, 18]. Bezogen auf die Prävention akuter Atemwegsinfekte ist zudem bekannt, dass Virusinfektionen in der Lunge die Aktivierung von Vitamin D in dessen aktiver Form (1,25-Dihydroxyvitamin D) fördern und damit antivirale Abwehrmechanismen (Cathelicidin) in Gang kommen [17, 18].

*Bezüglich Interventioneller Studien *zur Rolle einer Vitamin-D-Therapie bei COVID-19-Patienten sind zwei kleine Pilotstudien publiziert:

1. In einer randomisierten Interventionsstudie mit Calcifediol (Vitamin-D-Metabolit) wurde eine signifikante Verringerung des Bedarfs an Intensivstationsbehandlungen bei 76 Krankenhauspatienten mit einer belegten COVID-19-Lungenentzündung festgestellt. Während in der Kontrollgruppe 50 % intensivstationspflichtig wurden, waren es in der Calcifediolgruppe 2 % [19].
2. In einer placebokontrollierten Interventionsstudie mit 40 COVID-positiven Menschen mit keinen oder leichten Symptomen und einem belegten Vitamin-D-Mangel zeigte sich unter einer hoch dosierten Vitamin-D-Gabe, dass 63 % der Teilnehmer in der Interventionsgruppe und nur 21 % in der Kontrollgruppe vor Erreichen der 3. Woche COVID-RNA-negativ wurden [20].

Bisher fehlen große klinische Interventionsstudien, die die Rolle von Vitamin D bei COVID-19-erkrankten Menschen belegen. Eine große Studie bei COVID-19-erkrankten Menschen läuft derzeit in Boston (VIVID: https://clinicaltrials.gov/ct2/show/NCT04536298).

## Stellen die ersten DO-HEALTH-Resultate die aktuellen Empfehlungen zu 800 IE Vitamin D am Tag bei Erwachsenen im Alter 60+ in der Prävention von Frakturen infrage?

DO-HEALTH zeigte keinen Benefit von Vitamin D bezüglich des Risikos nichtvertebraler Frakturen. Wichtig ist jedoch, dass sich die Ergebnisse von DO-HEALTH auf die zusätzliche Einnahme von 2000 IE Vitamin D am Tag (zusätzlich zu der aktuellen Empfehlung von 800 IE am Tag) bei aktiven und gesunden älteren Menschen im Alter 70 und älter beziehen, die im überwiegenden Teil keinen Vitamin-D-Mangel hatten und in über 30 % der Fälle während der Studie 800 IE am Tag zusätzlich zur Studienmedikation einnahmen. Damit widersprechen die DO-HEALTH-Ergebnisse den aktuellen Empfehlungen von 800 IE Vitamin D am Tag für ältere Erwachsene nicht und stellen auch nicht die belegte Wirkung von 800 IE Vitamin D am Tag in der Prävention von Stürzen & Frakturen bei vulnerablen älteren Menschen mit Vitamin-D-Mangel und Osteoporose infrage [2–6].

Als wichtige Übereinstimmung bezüglich Vitamin-D-Empfehlungen für die Knochenbruchprävention, Sturzprävention und Prävention von akuten Atemwegsinfekten sollten Bolusgaben von Vitamin D vermieden werden und die tägliche Einnahme von Vitamin D bevorzugt stattfinden [5, 6].

### Zusammenfassend

fehlt bezüglich Vitamin D zum heutigen Zeitpunkt zwar der abschliessende Beweis einer grossen klinischen Studie bei COVID-19-erkrankten Patienten, jedoch sollte in der aktuellen Pandemiesituation die Evidenz bezogen auf die Senkung jeglicher akuter Atemwegsinfekte um 30 % mit der seitens des BAG seit 2012 bestehenden präventiven Empfehlung von 400 bis 1000 IE am Tag (bezogen auf die Knochengesundheit) eine ausreichende volksgesundheitliche Basis darstellen, um jetzt zu reagieren. Alternativ werden, bis die Studienergebnisse der Boston-VIVID-Studie vorliegen, Monate vergehen, in denen die sichere und günstige und breit verfügbare präventive Massnahme von 400 bis 1000 IE Vitamin D am Tag bereits greifen könnte. Dazu befinden wir uns in der Wintersaison, in der mindestens jeder zweite erwachsene Mensch in der Schweiz einen Vitamin-D-Mangel hat [21] und die COVID-19-Hochrisikopopulation von vulnerablen älteren Menschen das höchste Risiko für einen Vitamin-D-Mangel trägt [22].

## Empfehlung

1. Anhand dieser Risiko-Benefit-Abwägung in der aktuellen Pandemiesituation empfiehlt dieses Experten-Statement allen Menschen ab 60 Jahren täglich 800 IE Vitamin D nicht nur bezogen auf die Knochengesundheit, sondern in der aktuellen Pandemiesituation auch zur Prävention akuter Atemwegsinfekte. Diese Empfehlung bezieht sich prioritär auf die neuste Metaanalyse mit 42 Studien, welche für die tägliche Dosis von 400 bis 1000 IE Vitamin D eine Verminderung akuter Atemwegsinfekte um 30 % belegen konnte. Hausärzte sollten ihre Patienten jedoch darauf hinweisen, dass es bisher keinen Beleg gibt, dass Vitamin D auch vor akuten Atemwegsinfekten durch COVID-19 schützt, dass es jedoch zunehmende wissenschaftliche Hinweise gibt, dass die Behebung eines Vitamin-D-Mangels das Risiko von schweren Krankheitsverläufen der COVID-19-Atemwegsinfektion vermindern könnte.
  Konsistent mit den 2012 publizierten Empfehlungen des BAG bedarf die Supplementation mit 800 IE Vitamin D am Tag keiner vorherigen Testung des Blutspiegels, weil diese Dosierung auch bei Menschen ohne Mangel sicher ist und in 97 % der Fälle den Vitamin-D-Mangel sicher behebt.
2. Die Empfehlung von 800 IE Vitamin D am Tag sollte bei Menschen im Alter 60+ auch nach einer COVID-19-Impfung fortgeführt werden zur Prävention jeglicher akuten Atemwegsinfekte.

Aus volksgesundheitlicher Sicht einer entsprechenden Benefit-Risiko-Betrachtung erscheint es in der aktuellen und eskalierenden Pandemiesituation wichtig, diese Präventionsempfehlung zur Senkung des Risikos für akute Atemwegsinfekte schnell umzusetzen.

Zitat Editorial Lancet 1‑2021 zu Vitamin D und COVID-19: „Additional evidence could come in just too late. In an ideal world, all health decisions would be made based on overwhelming evidence, but a time of crisis may call for a slightly different set of rules.“

## Footnotes

1 Vorpublikation: https://www.medrxiv.org/content/10.1101/2020.07.14.20152728v3.
